# Comparative Evaluation of Responses from ChatGPT-5, Gemini 2.5 Flash, Grok 4, and Claude Sonnet-4 Chatbots to Questions About Endodontic Iatrogenic Events

**DOI:** 10.3390/healthcare13202615

**Published:** 2025-10-17

**Authors:** Makbule Taşyürek, Özkan Adıgüzel, Hatice Ortaç

**Affiliations:** 1 Department of Endodontics, Faculty of Dentistry, Dicle University, 21280 Diyarbakır, Türkiye; ozkanadiguzel@dicle.edu.tr; 2 Department of Biostatistics, Faculty of Medicine, Dicle University, 21280 Diyarbakır, Türkiye; haticeortac21@gmail.com

**Keywords:** artificial intelligence, large language models, ChatGPT, Gemini, Claude, Grok, iatrogenic events in endodontics

## Abstract

**Background:** The aim of this study was to compare four recently introduced LLMs (ChatGPT-5, Grok 4, Gemini 2.5 Flash, and Claude Sonnet-4). Experienced endodontists evaluated the accuracy, completeness, and readability of the responses given to open-ended questions about iatrogenic events in endodontics. **Methods:** Twenty-five open-ended questions related to iatrogenic events in endodontics were prepared. The responses of the four LLMs were evaluated by two specialist endodontists using a Likert scale for accuracy and completeness, and the Flesch Reading Ease Score (FRES), Flesch–Kincaid Grade Level (FKGL), Gunning Fog Index (GFI), Simplified Measure of Gobbledygook (SMOG), and Coleman–Liau Index (CLI) for readability. **Results:** The accuracy score of ChatGPT-5’s responses to open-ended questions (4.56 ± 0.65) was found to be significantly higher than those of Gemini 2.5 Flash (3.64 ± 0.95) and Claude Sonnet-4 (3.44 ± 1.19) (*p* = 0.009, and *p* = 0.002, respectively). Similarly, the completeness score of ChatGPT-5 (2.88 ± 0.33) was higher than those of Claude Sonnet-4, Gemini 2.5 Flash, and Grok 4 (*p* < 0.001, *p* = 0.002, and *p* = 0.007, respectively). In terms of readability measures, ChatGPT-5 and Gemini 2.5 Flash achieved better FRESs than Claude Sonnet-4 (*p* = 0.003, and *p* < 0.001, respectively). Conversely, FKGL scores were higher for Claude Sonnet-4 and Grok 4 compared to ChatGPT-5 (*p* < 0.001, and *p* = 0.008, respectively). Correlation analyses revealed a strong positive association (r_s_ = 0.77; *p* < 0.001) between accuracy and completeness, a weak negative correlation (r_s_ = −0.19; *p* = 0.047) between completeness and FKGL, and a strong negative correlation between (r_s_ = −0.88; *p* < 0.001) FKGL and FRES. Additionally, ChatGPT-5 demonstrated lower GFI and CLI scores than the other models, while its SMOG scores were lower than those of Gemini 2.5 Flash and Grok 4 (*p* = 0.001, and *p* < 0.001, respectively). **Conclusions:** Although differences were observed between the LLMs in terms of the accuracy and completeness of the responses, ChatGPT-5 showed the best performance. Even with high scores of accuracy (excellent) and completeness (comprehensive), it must not be forgotten that incorrect information can lead to serious outcomes in healthcare services. Therefore, the readability of responses is of critical importance, and when selecting a model, readability should be evaluated together with content quality.

## 1. Introduction

Artificial intelligence (AI) is a sub-branch of computer science which aims to develop intelligent systems that can undertake tasks that generally require human knowledge, such as learning, reasoning, and problem-solving [1]. By gaining experience and adapting to environments with new data, the performance of AI systems can be improved over time. Various AI applications can be used in dentistry to help diagnose, predict prognosis, develop telemedicine services, optimize the treatment process, reduce costs, support scientific research, and improve education [2].

In recent years, Large Language Models (LLMs) have rapidly infiltrated areas of medical research and clinical practice, transforming the field of AI [3]. LLMs, which benefit from their striking abilities in understanding and generating natural language and reasoning based on knowledge, are not limited to productively processing and synthesizing a large amount of medical knowledge, but also show significant potential in critical areas, such as supporting clinical decision-making processes, transforming medical education, and accelerating the discovery of scientific knowledge [4]. In dentistry, LLMs have significant potential for clinical application. As a result of processes such as automatic diagnosis, multimode analysis, personalized treatment planning, and patient education, the quality and productivity of diagnosis and treatment processes can be increased. However, there are still difficulties and limitations in the use of LLMs for clinical practice. In particular, in the diagnosis of complex cases and the formation of personalized treatment plans that require specialization, the responses to questions and decision support skills of these models have not yet reached a sufficient level [5].

In endodontics, LLMs can provide many benefits, especially in education. Some of these areas include summarizing research articles and clinical notes, accessing literature with natural language questions, extracting significant information from texts, and creating automatic reports based on clinical radiographic data. They can also provide real-time question-and-answer support for students and clinicians and provide patients with personalized information 24/7 on subjects such as treatment processes, postoperative instructions, drug use, and appointment planning [6]. The most recent versions of the most commonly used LLMs were used in this study.

Özden et al. reported that correct responses were only provided at a rate of 57.5% when using chatbots to answer questions related to dental trauma [7]. Suarez et al. compared the consistency and accuracy of responses to clinical questions in the field of endodontics given by ChatGPT compared to those of human specialists, and ChatGPT was found to reach an accuracy rate of 57.33% [8]. 

In another study by Qutieshat et al., the responses of students were compared with those of ChatGPT in the diagnosis of pulp and apical diseases. The results showed that ChatGPT obtained significantly higher accuracy (99.0%) than students (79.7%) and young clinicians (77.0%) [9]. Mohammad-Rahimi et al. evaluated the validity and reliability of the responses given by different AI chatbots to questions often asked about endodontics. The validity of the GPT-5 responses was found to be significantly higher than that of Google Bard and Bing [10]. Ekmekçi et al. evaluated the responses given by different AI applications to questions about regenerative endodontic procedures, and reported that ChatGPT-4 had the highest correct response rate (98.1%) and Gemini had the lowest rate (48%) [11].

Chat Generative Pre-Trained Transformer (ChatGPT; OpenAI, San Francisco, CA, USA) is a generative AI chatbot developed by OpenAI that has been on the market since 30 November 2022. It uses pretrained generative transformers, such as GPT-4o or o3, which can produce text, speech, and visuals as a response to user input [12,13]. Since January 2023, ChatGPT has been the most rapidly growing consumer software application in history and gained more than 100 million users within 2 months [14,15]. ChatGPT version 4.0 was introduced in March 2024, and version 4o was released in May 2024. This version, which included all the capabilities of GPT-3, offered enhanced accuracy, coherence, and depth; potential for more advanced tasks such as research assistance; more nuanced conversational abilities; integration into more complex systems; and special application potential in fields requiring expert knowledge, such as healthcare and finance. The manufacturer also stated that ChatGPT-4o showed a significant increase in processing speed, a decrease in delays, and notable improvements in both text and code processing, especially in languages other than English [16,17].

GPT-5, which was presented for use on 7 August 2025, was designed with a combined system architecture, said by the manufacturer to be formed from an intelligent and productive basic model, a deep-thinking mode, named GPT-5 Thinking, for difficult problems, and a directional component determining which mode is to be used in real time. Compared to previous versions, this model has provided a notable increase in speed and accuracy rates, and has been stated to have decreased the hallucination tendency, developed the ability to follow instructions, and minimized unnecessary confirmatory behavior. There has also been a reported significant improvement in performance in common areas of use, such as writing, encoding, and healthcare [18].

GPT-5, advancing from previous GPT models, presents a unified model architecture that incorporates real-time routing, improved reasoning, and significant enhancements in factual accuracy and multimodal functionality. GPT-5 demonstrates enhanced instruction following, increased reliability, and reduced hallucination rates across domains, including coding, healthcare, and writing. In the health domain, GPT-5 scored 46.2% on HealthBench Hard, significantly outperforming prior models [19].

According to the manufacturer, GPT-5 is the best model developed in the field of healthcare, supporting the raising of awareness among users about healthcare. GPT-5, which clearly outperformed previous models in the HealthBench performance evaluation, first indicated potential problems and addressed questions to provide more useful answers. Although it cannot replace a medical specialist, it is helpful for interpreting results, asking the right questions, and making informed decisions. It has also been reported that, as GPT-5 contains complex and open-ended questions, it significantly reduces the hallucination rate in evaluations where factual accuracy is mandatory (e.g., LongFact and FActScore) [18].

Google Bard is an AI developed by Google, supported initially by the Language Model for Dialogue Applications (LaMDA), which is part of Google’s own LLM family, and later by the Pathways Language Model (PaLM) 2 LLM. Google Bard was launched on the market on 21 March 2023, and then was renamed Gemini in February 2024 (Google, San Francisco, CA, USA). It is an AI-supported information acquisition tool and advanced chatbot that uses a native multimodal model that can productively analyze and adapt many data formats, such as text, sound, and video. It can be used free of charge, and an unlimited number of questions can be asked or dialogs formed. Gemini offers free dialog AI services using a series of deep learning algorithms to answer questionnaires [20,21].

Gemini 2.5 was introduced on 26 March 2025. According to the manufacturer, it is a thinking model designed to solve increasingly complex problems. The Gemini 2.5 models can pass thoughts through a logic filter before responding and are therefore considered to provide advanced performance and increased accuracy [19].

While the Gemini model has the ability to give comprehensive and informed responses to questions, ChatGPT models can produce creative and consistent texts [22,23]. Both ChatGPT and Gemini are trained on the interactive learning principle. Therefore, the accuracy and creativity of the information produced can be improved through human feedback. As a result of this process, the model develops responses over time and exhibits better performance [24].

Claude (Anthropic, San Francisco, CA, USA) is an LLM that was designed with particular emphasis on ethical and security issues, adopting a more controlled and transparent approach. It was launched on the market in March 2023 [25]. Claude 3 Opus and Claude 3.5 Sonnet can read and analyze not only textual input but also visual data. It therefore has the ability to deeply understand and interpret both written and visual content [26]. Claude 4, a version containing Opus and Sonnet, was launched on the market in May 2025. By presenting a broad contextual window and low hallucination rates, Claude Sonnet-4 is ideal for answering questions regarding large knowledge bases, documents, and code bases [27].

On 4 November 2023, the X AI company of Elon Musk (San Francisco, CA, USA) introduced a new AI model named Grok. The ability to respond to user commands using “real-time information” from the X platform (previously Twitter) differentiates Grok from previous LLMs. Grok defines itself as a strong research assistant that rapidly presents information, processes data, creates new ideas, and can recommend questions. According to the manufacturer, Grok 4, which was introduced on 1 July 2025, was trained to use tools with reinforcement learning. In this way, it may support the thinking process with tools such as code interpretation and web screening in situations that are generally difficult for LLMs. When searching for real-time knowledge or responding to complex research questions, Grok 4 selects its own search questions, thereby forming high-quality responses by collecting information from the web in general and, when necessary, examining it in depth. In particular, Grok 4 Heavy stands out as an exceptionally challenging benchmark designed to simulate PhD-level problem solving [28].

In the light of this information, the four LLMs selected for use in this study were the current versions of ChatGPT-5, Grok 4, Gemini 2.5 Flash, and Claude Sonnet-4.

A significant number of adverse events or accidents may occur during endodontic treatment. Since 2002, these have been accepted as “unfortunate events due to attention not paid to some details and sometimes completely unforeseen circumstances occurring during treatment”. These types of events can occur at any stage of endodontic treatment and have the potential to lead to treatment failure. Walton and Torabinejad defined endodontic errors as “unwanted or unforeseen situations during root canal treatment, which can affect the prognosis” [29].

Hypochlorite accidents lead to more extensive endodontic complications caused by apical extrusions, such as emphysema, sinusitis, and nerve damage (paresthesia and anesthesia). Emphysema is defined as the entry of air or other gases below the skin and submucosa. Consequently, gas accumulation develops in the spaces between tissues, for example, with the accidental injection of air or oxygen expressed by hydrogen peroxide, which can lead to severe complications. If the filling material is injected into sensitive areas, such as the maxillary sinus, inferior alveolar nerve within the mandibular canal, or mental foramen, more severe and even potentially irreversible damage can occur, including maxillary sinusitis, Aspergillus infections, paresthesia, dysesthesia, and similar neurological complications [30,31].

In situations where rapid and correct decisions are of vital importance, AI can provide significant support to dentists. By minimizing human error in the decision-making process, the clinician’s workload is lightened, thereby allowing higher quality, reliable, and consistent treatment to be offered to the patient [32]. Continuous access meets the need for learning at the right time, especially in situations where rapid access to information is of vital importance in clinical settings [33]. For example, when a student is preparing to perform a canal treatment procedure, a chatbot can be quickly asked to explain step-by-step guides and certain techniques. Additionally, to meet different learning preferences, advanced chatbots may integrate various media types to develop the learning experience [34]. Chatbots have been examined in terms of developing the learning experience, assisting in the clinical decision-making process, and the potential to support various aspects of the dentistry syllabus [35].

Iatrogenic events in endodontics are critical situations that can lead to serious health problems. Every dentist may encounter these types of situations throughout their professional life. However, the fact that these events are not frequently seen by every clinician may be a reason for the lack of clinical experience and delay in emergency intervention. This can negatively affect both the general health status of the patient and the success of root canal treatment. Although many studies have examined the use of chatbots in different branches of healthcare, there are insufficient studies examining the approaches to iatrogenic events occurring in endodontics.

To the best of our knowledge, no previous study has investigated the use of the ChatGPT-5 AI model related to iatrogenic events in endodontics. The aim of this study was to compare four recently introduced LLMs (ChatGPT-5, Grok 4, Gemini 2.5 Flash, and Claude Sonnet-4) through evaluations by experienced endodontists of the accuracy, completeness, and readability of the responses given to open-ended questions specifically prepared in relation to iatrogenic events in endodontics.

The null hypothesis of the study was that there would be no statistically significant difference between ChatGPT-5, Grok 4, Gemini 2.5 Flash, and Claude Sonnet-4 LLMs in terms of approach performance, accuracy, completeness, and readability of the responses to questions related to iatrogenic events in endodontics.

## 2. Materials and Methods

As this study did not involve human or animal subjects, ethical approval was not required. A total of 52 questions were prepared in an open-ended format by two endodontic specialists, maintaining scientific accuracy and clinical importance at the forefront. The questions were developed based on “Managing Iatrogenic Events”, which is the 20th chapter of Cohen’s *Pathways of the Pulp* (12th edition) [36].

To improve the clarity of the questions and the evaluation process, an expert panel was established. This panel included two experienced endodontists and two general dentists, each of whom reviewed the questions in terms of clarity, representativeness, and clinical relevance. The final set of 25 questions, prepared to enhance content validity, was subjected to an informal pilot review by the expert panel. At this stage, no formal content validity index or inter-rater reliability coefficients were calculated. Panel members evaluated each question in a structured but flexible process through iterative discussions and consensus, assessing the clarity, comprehensiveness, and clinical relevance of the items; they agreed on necessary revisions to ensure content integrity.

This approach offered a more practical and rapid method compared to formal measurement methods and was also found to be sufficient to ensure the clinical validity of the items. Therefore, it was not deemed necessary to apply a content validity test at this stage. The panel’s verbal and consensus-based contributions ensured that the set of questions appropriately covered the fundamental dimensions of iatrogenic events in endodontics.

Each question was evaluated in a paired format of “acceptable” (understandable at a level to allow explanation of the subject) or “unacceptable” (an insufficient level of clarity of the question so that the aim of the subject cannot be expressed completely and clearly). A question pool of 52 open-ended questions was formed at the beginning of the study, and the 25 most appropriate questions were selected for inclusion in the analyses. The selected questions are listed in Table 1.

The questions were asked in accounts newly created by a single researcher in the ChatGPT-5 Thinking mode (Open AI, https://chatgpt.com/ (accessed on 8 August 2025), Gemini 2.5 Flash (Google, https://gemini.google.com/ (accessed on 8 August 2025), Claude Sonnet-4 (Anthropic, https://claude.ai/new (accessed on 9 August 2025), and Grok 4 Heavy mode (X.AI, https://x.ai/grok (accessed on 9 August 2025) platforms. The questions were asked at similar time intervals within a 24 h timeframe between 8 and 9 August 2025. Access was carried out solely through the web interfaces of the relevant platforms via a computer and was designed to reflect the experience most closely resembling real clinical conditions. LLMs were not specifically trained regarding the Cohen’s *Patways of the Pulp* (12th edition) book, which was used to prepare the questions. No pre-tests, prompt optimizations, or additional interventions were applied. Interactions with the models were limited exclusively to predetermined English questions prepared for research purposes. The English language was used for both the input and the output. Apart from this, no further instructions, interventions, or alternative forms of use were involved. No generation parameters (including temperature or sampling settings) were modified by the investigators. Accordingly, all responses were produced under the default system configurations, reflecting typical real-world usage conditions.

This study does not aim to examine the consistency of the models. The study was designed to compare the models’ ability to accurately and completely answer single questions related to sudden, iatrogenic endodontic events that dentists may encounter in clinical practice, which is why all questions were asked only once. This approach aimed to evaluate the model’s instantaneous response performance to an unexpected, impulsive question that a dentist might pose to an AI chatbot.

To eliminate the possibility of the responses of the AI-based language models being affected by previous questions and answers, each question was asked in a separate chat session. By clearing the system memory at the start of the session, the previous chat was completely erased, enabling the model to focus only on the current input. This approach aimed to minimize contextual guidance or predictive risks that could emerge as a result of consecutive interactions. Thus, it was ensured that each response produced was independent, unbiased, and based only on the current question. In addition, each language model was allowed to provide only one response, and was prevented from correcting or reproducing the first response after it was produced. All the responses obtained were recorded in Microsoft Word format (Microsoft, Redmond, WA, USA).

The responses obtained from the four different AI models were anonymized and then presented to two experienced endodontic specialists. To ensure the impartiality of the evaluation process, the information about which model the responses belonged to was concealed. The responses of each chatbot were color-coded before being presented to the researchers. This allowed the researchers to examine the responses without knowing which chatbot they belonged to.

The 20th chapter of Cohen’s *Pathways of the Pulp* (12th edition), “Managing Iatrogenic Events”, was used as the reference standard in the evaluation process [36]. This reference text was presented to the expert evaluators along with anonymized responses. To provide a structured and consistent scoring process, each evaluator used a standardized Excel scoring table (Microsoft, Redmond, WA, USA).

In the evaluation performed by the specialists, the responses were analyzed in two main dimensions: content accuracy and completeness.

A five-point Likert scale was used to evaluate the accuracy of the responses provided by the LLMs [37]. The evaluation process was conducted in a double-blind design to minimize the risk of bias.

**Five-Point Likert Scale (for evaluating accuracy):**

1 = Very Poor: Weak accuracy with no flow, most information missing.
2 = Poor: Generally weak accuracy; some information provided but major gaps remain.
3 = Fair: Moderate accuracy; some important details included, but others missing.
4 = Good: Good accuracy and flow; most relevant information is present.
5 = Excellent: Extremely good accuracy and flow; all information is complete and well-organized.

To evaluate the completeness of the information in the responses provided by the LLMs, a 3-point Likert scale was used [37]. The evaluation process was conducted in a double-blind design to minimize the risk of bias.

**Three-Point Likert Scale (for evaluating completeness):**

1 = Incomplete: Response only addresses some aspects of the question, missing key elements.
2 = Adequate: Response covers all aspects of the question with the necessary information for completeness.
3 = Comprehensive: Response covers all aspects of the question and goes beyond expectations by providing extra context or information.

The levels of readability of the responses were evaluated using the Flesch Reading Ease Score (FRES) and Flesch–Kincaid Grade Level (FKGL) indexes. The measurements were performed using an open-access online calculation tool (https://goodcalculators.com/flesch-kincaid-calculator (accessed on 10 August 2025)) [38]. The FRES and FKGL indexes express the difficulty level of texts numerically. The calculation of readability is based on average sentence length (the average number of words per sentence) and average word length (the average number of syllables per word). The FRES and FKGL values were obtained using the standard formulae defined in the literature [39].

*FRES: 206.835 − 1.015 × (total words/total sentences) − 84.6 × (total syllables/total words)* [38].

*FKGL: 0.39 × (total words/total sentences) + 11.8 × (total syllables/total words) − 15.59* [38].

The responses of each LLM were evaluated using the online tool from https://readable.com to calculate the Simplified Measure of Gobbledygook (SMOG), Coleman–Liau Index (CLI), and Gunning Fog Index (GFI) scores.

SMOG is regarded as the gold standard for assessing the readability of health education materials, as it predicts the level of literacy required for 100% comprehension of texts [40].

*SMOG: Grade level = 1.0430 × √[Number of polysyllabic words × (30/Number of sentences)] + 3.1291 [41].*

CLI: This index is used to determine the readability of a text. It takes into account the average number of letters per 100 words and the average sentence length. The resulting score indicates the U.S. school grade level required to understand the text. Lower scores suggest that the text is easier to read, while higher scores mean that it requires more advanced reading skills [42].

*CLI = (0.0588 × L) − (0.296 × S) − 15.8 [41].*

*L = average number of letters per 100 words.*

*S = average number of sentences per 100 words.*

GFI: This index was developed to measure the comprehensibility of a text and typically produces a score between 6 and 17. The resulting value indicates the estimated education level of the reader: a score of 6 corresponds to the level of an 11–12-year-old student, 12 represents high school graduation level, and 17 corresponds to a university graduate level. Lower scores suggest that the text is simpler and easier to read, while higher scores indicate a more complex reading level [43].

GFI = 0.4 × [(total words/total sentences) + 100 × (complex words/total words)] [41].

For the calculation of readability indices, only the response part of each question was individually entered into the online tools (goodcalculators.com and readable.com). In this way, an independent readability score was obtained for each question. These scores were then recorded in an Excel spreadsheet (Microsoft, Office 365, Redmond, WA, USA) and compiled for use in the statistical analyses. This process allowed for the readability of each model’s responses to be evaluated individually, followed by aggregate analyses based on mean and distribution values.

**Figure 1:**
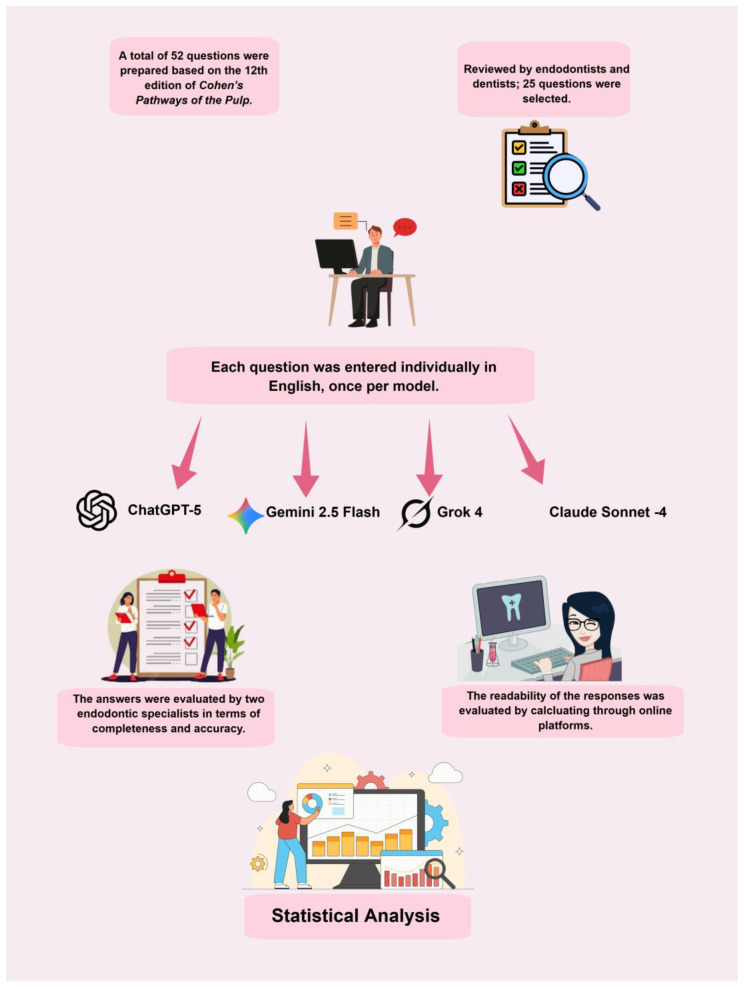
Overview of the workflow for research design and evaluation.

FRES values range between 0 and 100 points, with higher scores indicating an increase in text readability. The FRESs can be converted to estimated education levels using standardized tables. The FKGL value indicates the minimum education level required to read a text easily. Lower FKGL values indicate that a lower education level is sufficient to understand the text. It is recommended that documents containing health information have a readability level of 8th grade or lower [44] (Table 2). The comprehensive workflow of the study is shown in Figure 1.

### Statistical Analysis

To examine the agreement between evaluators, the Intraclass Correlation Coefficient (ICC) values were calculated separately for the accuracy and completeness scores. The conformity of continuous variables to a normal distribution was assessed using the Shapiro–Wilk test. Variables showing normal distribution are stated as mean ± standard deviation (SD) values, and those not conforming to normal distribution are stated as median (minimum–maximum) values. According to the normality test results, the ANOVA test was used to compare groups when there were more than two groups and a normal distribution was observed, while the Kruskal–Wallis test was used when a normal distribution was not observed. Following the ANOVA test, subgroup analyses were performed using the Bonferroni test when overall significance was present. Following the Kruskal–Wallis test, subgroup analyses were performed using the Dunn–Bonferroni test when overall significance was present.

The relationships between scores were examined using correlation analysis, and Pearson and Spearman correlation coefficients were calculated.

Effect sizes for between-group comparisons were calculated using eta-squared (η^2^) for both parametric and non-parametric analyses in order to evaluate the practical significance of the observed differences.

SPSS for Windows, version 27.0 (IBM Corp., Armonk, NY, USA), was used for all statistical analyses. A type I error of 5% was accepted, and the level of statistical significance was set at *p* < 0.05.

## 3. Results

According to the ICC analysis (C, 1), the evaluator consistency of all models was statistically significant (*p* < 0.001). ChatGPT-5 showed high agreement (ICC = 0.891, 95% CI: 0.768–0.950), Gemini 2.5 Flash (ICC = 0.952) and Grok 4 (ICC = 0.950) showed excellent agreement, and Claude Sonnet-4 showed the highest and almost perfect agreement (ICC = 0.986). According to the ICC analysis (C, 1), the inter-evaluator agreement in the completeness points of all the models was found to be statistically significant (*p* < 0.001). A high level of inter-evaluator consistency was observed in ChatGPT-5 (ICC = 0.840), Gemini 2.5 Flash (ICC = 0.929), Grok 4 (ICC = 0.948), and Claude Sonnet-4 (ICC = 0.966).

**Figure 2:**
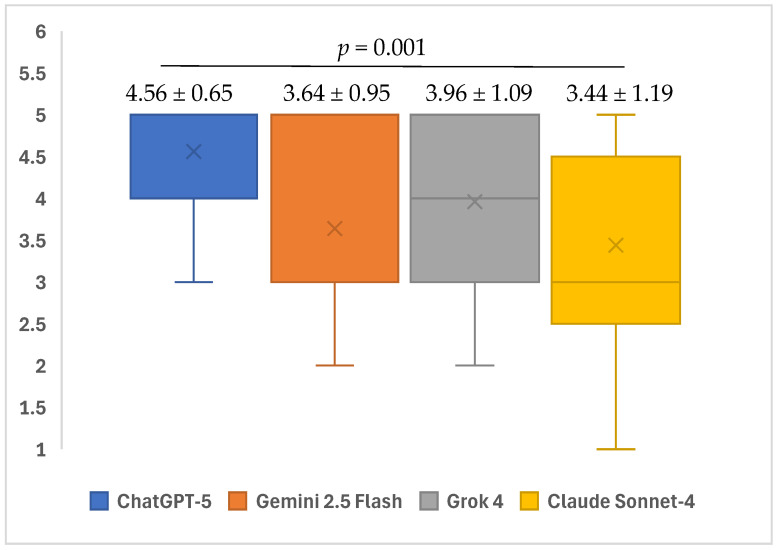
Mean accuracy scores of ChatGPT-5, Gemini 2.5 Flash, Grok 4, and Claude Sonnet-4 in responding to questions related to iatrogenic events in endodontics.

A statistically significant difference was found between the four different AI models in terms of the accuracy scores of the responses to the open-ended questions related to iatrogenic events in endodontics (*p* = 0.001). In the subgroup analyses, ChatGPT-5’s responses to open-ended questions showed a higher mean accuracy score with a median of 5 (minimum: 3–maximum: 5) compared to Gemini 2.5 Flash (median 3, minimum: 2–maximum: 5) and Claude Sonnet-4 (median 3, minimum: 1–maximum: 5). These differences were statistically significant (*p* = 0.009 and *p* = 0.002, respectively). No statistical significance was observed in the other paired group comparisons (*p* > 0.05) (Table 3) (Figure 2).

**Figure 3:**
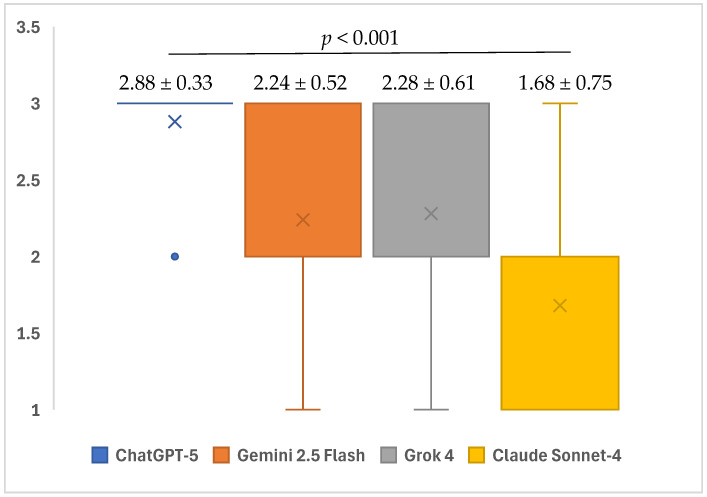
Mean completeness scores of ChatGPT-5, Gemini 2.5 Flash, Grok 4, and Claude Sonnet-4 in response to questions related to iatrogenic events in endodontics.

A statistically significant difference was determined between the four different AI models in terms of the completeness levels of the responses given to the open-ended questions related to iatrogenic events in endodontics (*p* = 0.001). In the subgroup analyses, the median completeness scores of ChatGPT-5 (3; minimum: 2–maximum: 3) and Grok 4 (2; minimum: 1–maximum: 3) for open-ended questions were found to be significantly higher than those of Claude Sonnet-4 (2; minimum: 1–maximum: 3) (*p* < 0.001 and *p* = 0.046, respectively). Furthermore, the median score of ChatGPT-5 (3; minimum: 2–maximum: 3) for open-ended questions was significantly higher compared to those of Gemini 2.5 Flash (2; minimum: 1–maximum: 3) and Grok 4 (2; minimum: 1–maximum: 3) (*p* = 0.002 and *p* = 0.007, respectively). No statistical significance was observed in the other paired group comparisons (*p* > 0.05) (Table 3) (Figure 3).

**Figure 4:**
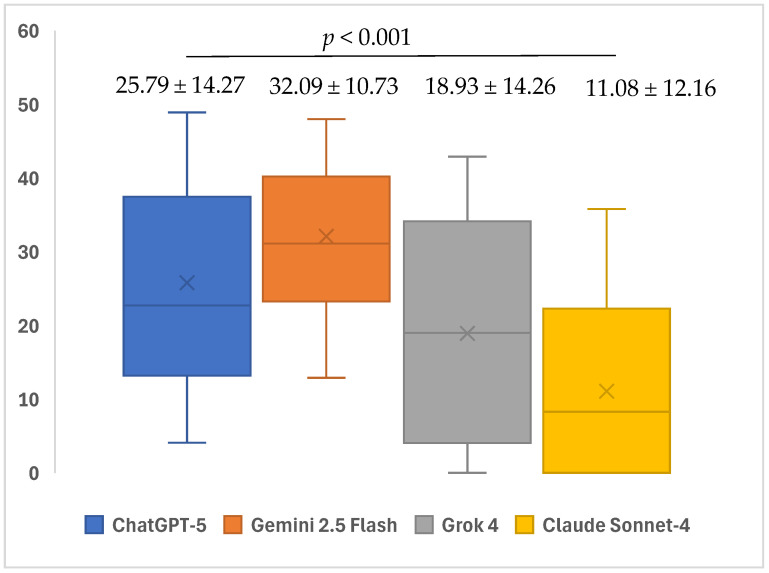
Mean readability FRESs of ChatGPT-5, Gemini 2.5 Flash, Grok 4, and Claude Sonnet-4 in response to questions related to iatrogenic events in endodontics.

According to the FRES points, a statistically significant difference was observed between the AI models in terms of the readability levels of the responses given to the open-ended questions (*p* < 0.001). In the subgroup analyses, the median FRES readability scores of ChatGPT-5 (22.7; minimum: 4.1–maximum: 48.9) and Gemini 2.5 Flash (31.1; minimum: 12.9–maximum: 48) for open-ended questions were found to be significantly higher than those of Claude Sonnet-4 (8.3; minimum: 0–maximum: 35.8) (*p* = 0.003 and *p* < 0.001, respectively). Furthermore, the median FRES readability scores of Gemini 2.5 Flash (31.1; minimum: 12.9–maximum: 48) were significantly higher than those of Grok 4 (19; minimum: 0–maximum: 42.9) (*p* = 0.011). No significant differences were observed in the other pairwise comparisons (*p* > 0.05) (Table 3) (Figure 4).

**Figure 5:**
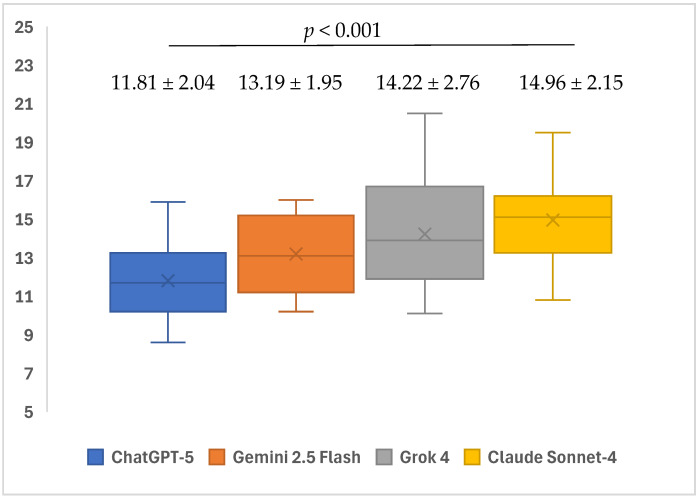
Mean readability FKGL scores of ChatGPT-5, Gemini 2.5 Flash, Grok 4, and Claude Sonnet-4 when responding to questions related to iatrogenic events in endodontics.

There was a statistically significant difference between the FKGL points of the different models (*p* < 0.001). In the subgroup analyses, the median FKGL readability scores of Claude Sonnet-4 (15.1; minimum: 10.8–maximum: 19.5) and Grok 4 (13.9; minimum: 10.1–maximum: 20.5) for open-ended questions were found to be significantly higher than those of ChatGPT-5 (11.7; minimum: 8.6–maximum: 15.9) (*p* < 0.001 and *p* = 0.008, respectively). No significant differences were observed in the other pairwise comparisons (*p* > 0.05) (Table 3) (Figure 5).

**Figure 6:**
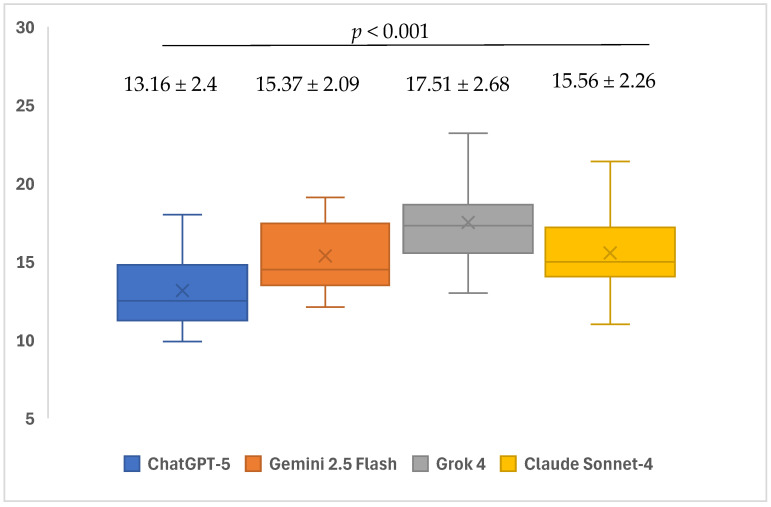
Mean readability-GFI scores of ChatGPT-5, Gemini 2.5 Flash, Grok 4, and Claude Sonnet-4 in response to questions related to iatrogenic events in endodontics.

The readability level of the AI models’ responses to open-ended questions, as indicated by GFI scores, showed significant differences (*p* < 0.001). Within the subgroup analyses, the average GFI scores of ChatGPT-5 (13.16 ± 2.4) responses to open-ended questions were found to be significantly lower than the average scores of Gemini 2.5 Flash (15.37 ± 2.09), Grok 4 (17.51 ± 2.68), and Claude Sonnet-4 (15.56 ± 2.26) models (*p* = 0.008, *p* < 0.001, and *p* = 0.003, respectively). The average GFI scores of the responses given by the Grok 4 (17.51 ± 2.68) model to open-ended questions were found to be significantly higher than the average scores of the Gemini 2.5 Flash (15.37 ± 2.09) and Claude Sonnet-4 (15.56 ± 2.26) models (*p* = 0.011 and *p* = 0.007). No significant differences were found in the other pairwise group comparisons (*p* > 0.05) (Table 3) (Figure 6).

**Figure 7:**
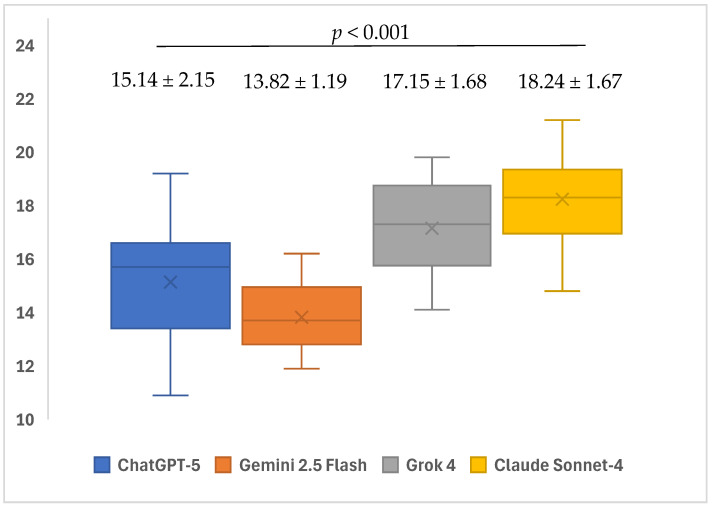
Mean readability CLI scores of ChatGPT-5, Gemini 2.5 Flash, Grok 4, and Claude Sonnet-4 in response to questions related to iatrogenic events in endodontics.

The readability levels of AI models’ responses to open-ended questions show significant differences based on CLI scores (*p* < 0.001). Within subgroup analyses, it was determined that the average CLI score of ChatGPT-5 (15.14 ± 2.15) model’s responses to open-ended questions was significantly lower than the average scores of Grok 4 (17.15 ± 1.68) and Claude Sonnet-4 (18.24 ± 1.67) models (*p* < 0.001 and *p* < 0.001, respectively). The average CLI score of the Gemini 2.5 Flash (13.82 ± 1.19) model’s responses to open-ended questions was found to be significantly lower than the average scores of the ChatGPT-5 (15.14 ± 2.15), Grok 4 (17.15 ± 1.68), and Claude Sonnet-4 (18.24 ± 1.67) models (*p* = 0.046, *p* < 0.001, and *p* < 0.001, respectively). No significant differences were found in the other pairwise group comparisons (*p* > 0.05) (Table 3) (Figure 7).

**Figure 8:**
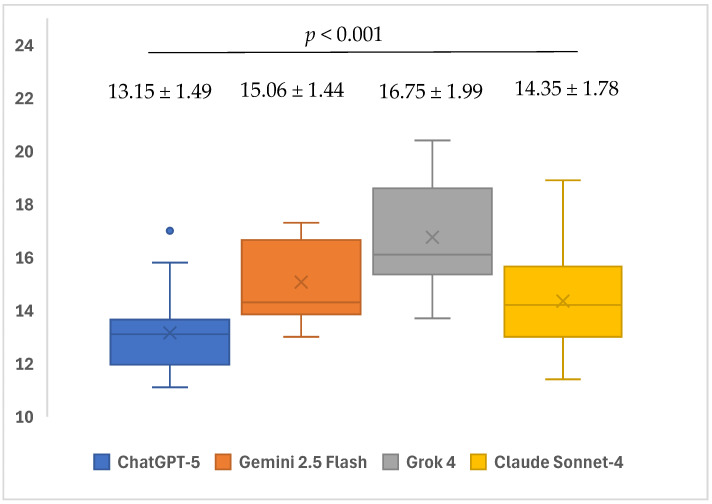
Mean readability-SMOG scores of ChatGPT-5, Gemini 2.5 Flash, Grok 4, and Claude Sonnet-4 in response to questions related to iatrogenic events in endodontics.

The readability levels of the AI models’ responses to open-ended questions showed significant differences based on SMOG scores (*p* < 0.001). Within subgroup analyses, it was determined that the average SMOG score of ChatGPT-5’s (13.15 ± 1.49) responses to open-ended questions was significantly lower than the average scores of Gemini 2.5 Flash (15.06 ± 1.44) and Grok 4 (16.75 ± 1.99) (*p* = 0.001 and *p* < 0.001, respectively). The mean SMOG score of the responses given by the Grok 4 (16.75 ± 1.99) model to open-ended questions was significantly higher than the mean score of the responses given by the Claude Sonnet-4 (14.35 ± 1.78) model (*p* = 0.001). No significant differences were found in the other pairwise group comparisons (*p* > 0.05) (Table 3) (Figure 8).

In addition to statistical significance, eta squared (η^2^) was calculated to assess the magnitude of the differences between groups. For accuracy, η^2^ = 0.13 indicated a magnitude close to a large effect, while for completeness, η^2^ = 0.34 revealed a very strong effect size. These findings demonstrate that the differences among models in terms of accuracy and completeness are not only statistically significant but also practically/operationally meaningful.

To further evaluate the magnitude of differences across models, eta squared values were calculated for each of the five readability indices. The results were η^2^ = 0.19 for FKGL, η^2^ = 0.26 for FRES, η^2^ = 0.26 for GFI, η^2^ = 0.51 for CLI, and η^2^ = 0.38 for SMOG. These values indicate that all readability indices exhibited large effect sizes, with the CLI in particular pointing to a very strong effect. These findings reveal substantial differences in readability levels among the evaluated AI models and highlight the practical importance of these indices in assessing the comprehensibility of model outputs for users.

A significant positive correlation was found between accuracy score and completeness score (r_s_ = 0.77; *p* < 0.001). The findings indicate that an increase in the accuracy score is associated with an increase in the completeness score. A significant negative correlation was found between completeness score and FKGL (r_s_ = −0.19; *p* = 0.047). As the FKGL value increases, the completeness score tends to decrease. A significant negative correlation was also found between completeness score and SMOG (r_s_ = −0.20; *p* = 0.045). As the SMOG value increases, the completeness score tends to decrease (Table 4).

A significant negative correlation was found between FKGL and FRES readability scores (r_s_ = −0.88; *p* < 0.001). As the FKGL value increases (indicating higher reading difficulty), the FRES value decreases (indicating lower ease of reading). A significant positive correlation was found between FKGL and GFI readability scores (r_s_ = 0.81; *p* < 0.001), showing that an increase in FKGL scores is associated with an increase in GFI scores. A significant positive correlation was also found between FKGL and CLI readability scores (r_s_ = 0.76; *p* < 0.001), indicating that an increase in FKGL scores is associated with an increase in CLI scores. Similarly, a significant positive correlation was found between FKGL and SMOG readability scores (r_s_ = 0.72; *p* < 0.001), showing that an increase in FKGL scores is associated with an increase in SMOG scores (Table 5).

A significant negative correlation was found between FRES and GFI readability scores (r_s_ = −0.59; *p* < 0.001). As the FRES value increases, the GFI value decreases. A significant negative correlation was also found between FRES and CLI readability scores (r_s_ = −0.89; *p* < 0.001). As the FRES value increases, the CLI value decreases. Furthermore, a significant negative correlation was found between FRES and SMOG readability scores (r_s_ = −0.45; *p* < 0.001). As the FRES value increases, the SMOG value decreases (Table 5).

A significant positive correlation was found between GFI and CLI readability scores (r_s_ = 0.58; *p* < 0.001), indicating that an increase in GFI scores is associated with an increase in CLI scores. A significant positive correlation was also found between GFI and SMOG readability scores (r_s_ = 0.94; *p* < 0.001), showing that an increase in GFI scores is associated with an increase in SMOG scores. Finally, a significant positive correlation was found between CLI and SMOG readability scores (r_s_ = 0.46; *p* < 0.001), indicating that an increase in CLI scores is associated with an increase in SMOG scores (Table 5).

## 4. Discussion

Root canal treatment consists of four main stages. First, an accurate diagnosis is established through clinical evaluation and, when necessary, radiographic examination. Second, an access cavity is prepared to allow entry into the root canal system. In the third stage, the canals are thoroughly cleaned and shaped using specialized instruments and irrigating solutions with an activation system (sonic, ultrasonic, laser, etc.). Finally, the obturation phase involves sealing the canals with biocompatible materials to ensure hermetic closure and prevent reinfection [45,46,47,48,49]. The success of each step depends on the correct application. The overall quality of the procedure is increased by performing all these four steps in the best way and by having sufficient knowledge of potential procedural errors that could occur at each stage. Therefore, knowledge of the most frequently observed procedural errors is of the greatest importance in preventing them [50,51].

The practical clinical application of LLMs currently faces many difficulties and limitations. For example, there is not yet an optimal level of ability to respond to specialized questions in the field of dentistry, especially in the diagnosis of complex cases and the formation of personalized treatment plans [5].

It is thought that LLMs will be able to accelerate decision-making for intervention in iatrogenic events in endodontics and will be able to provide dentists with high-quality, consistent, and reliable medical support in the processes of correct diagnosis and effective intervention in high-risk conditions. Therefore, this study aimed to evaluate four different LLMs in terms of the accuracy, completeness, and readability of their responses to questions concerning the clinical management of endodontic iatrogenic events that require knowledge, care, and prompt action.

Large language models, such as ChatGPT, Gemini, Grok, and Claude, play important emerging roles in health support, but also have significant drawbacks. ChatGPT is extensively studied and utilized for patient education, summarizing clinical information, and drafting documentation; however, the potential for hallucinations remains a concern [52]. Gemini and Claude have been included in multimodal diagnostic benchmarks with competitive accuracy, although have received less attention [53]. While continually bettering themselves, Grok and other LLMs are revolutionizing medicine [54].

Chatzopoulos et al. observed that there is still a need for the development of LLMs in terms of the comprehensiveness, scientific validity, logical consistency, and clear presentation in the responses given to open-ended clinical questions [55]. This is especially evident in complex clinical conditions, which are characterized by multifactorial interactions and significant subjectivity, as LLMs tend to have incomplete or biased information in such conditions [5]. Iatrogenic complications that can be encountered in endodontics represent an example of clinical conditions that may require clinicians to direct open-ended questions to LLMs. Therefore, to evaluate the capacity of LLMs to correctly and comprehensively respond to open-ended questions in this study, the questions were structured to be open-ended and appropriate to the potential knowledge requirements of clinicians.

Limiting the interaction to single questions allowed for a clearer evaluation of the capabilities of the LLMs to provide direct, specific, and relevant responses to complex questions without the need for redirection. This approach also allowed for the comparison of simultaneous responses from a clinical perspective. Similarly, the questions in a previous study that examined the performance of LLMs in the clinical decision-making process in endodontics were directed to be answered only once without re-phrasing, with the aim of simulating a real clinical consultation [56].

In the study by Sezer and colleagues comparing the performance of advanced artificial intelligence models in pulp therapy for immature permanent teeth, no modifications, pre-testing, or prompt refinements were applied to the questions, thereby ensuring that the study conditions closely reflected real-world usage. Similarly, in our study, no additional prompts were provided for the questions, taking into account the fact that iatrogenic events in endodontics generally occur unexpectedly and that clinicians tend to directly query the incident when confronted with such situations [57].

Although only a few studies have directly examined the completeness of LLM responses to open-ended questions related to iatrogenic events in endodontics, this criterion has been addressed in various studies in other areas of dentistry. Molena et al. evaluated the accuracy and completeness of ChatGPT responses to questions formulated by specialists. In terms of completeness, the median value was 2.00, and the mean value was 2.07 on a scale of 3 [58]. In a study by Gurbuz et al., the accuracy and completeness of ChatGPT-4o were evaluated in the treatment of cases with cervical lesions and no decay using a 3-point Likert scale. Regarding completeness, different results were produced from the three areas; two cases obtained three points in diagnosis, no comprehensive response was obtained for clinical management in any case, and one case obtained three points in the area of surgical management [59]. Similarly, in the current study, the extent of completeness of the responses produced by LLMs was defined using a 3-point Likert scale based on the information in the “Managing Iatrogenic Events” section, which is the 20th chapter of the 12th edition of Cohen’s *Pathways of the Pulp*.

In a previous study that evaluated the performance of Google Bard, ChatGPT-3.5, and ChatGPT-4 in the clinical decision-making process in endodontics, the highest accuracy and completeness points and the most accurate information were obtained from ChatGPT-4, followed by ChatGPT-3.5 and Google Bard [56]. The earlier AI model of Google Bard underwent significant developments and was then introduced as Gemini in December 2023, with noteworthy advances made in image and video processing capabilities in particular, and thus the correct response rate was increased [60]. Büker et al. compared the performances of LLMs in endodontic clinical decision support, and reported that ChatGPT-4.0 displayed superior performance, whereas Gemini 2.0 Flash and ChatGPT-3.5 showed overall accuracy at a similar level [61]. A similar pattern was observed in the current study. When more recent versions were compared, ChatGPT-5 was determined to show better performance in terms of both accuracy and completeness than Gemini 2.5 Flash. Despite all these developments, the most recent version of Gemini 2.5 Flash was unable to reach the level of the most recent ChatGPT version, ChatGPT-5.

In another study conducted by pediatric dentists in compliance with the IADT guidelines, the responses of four chatbots to 25 open-ended questions related to traumatic dental injuries in the deciduous dentition period were evaluated. Although no statistically significant difference was determined between the ChatGPT-4o, Claude 3.7, and Gemini Advanced models in terms of accuracy, ChatGPT-4o obtained the highest overall accuracy score [62]. Comprehensive comparisons of the National Board of Medical Examiners sample questions have shown that GPT-4 has shown better performance more consistently than other LLMs in various medical specialist areas. While GPT-4 reached an accuracy rate of 100%, this rate was 82.2% for ChatGPT-3.5, 84.7% for Claude, and 75.5% for Google Bard [63]. Similarly, in the current study, both the accuracy and completeness points of the responses given to open-ended questions by ChatGPT-5 were found to be higher than those of Gemini 2.5 Flash and Claude Sonnet-4.

In a study comparing the accuracy and completeness performances of LLMs in treatment planning for restorations of teeth that have undergone endodontic treatment, Shirani et al. reported that Gemini 2.5 Pro and Claude 3.7 Sonnet provided more complete and accurate restorative treatment planning responses compared to ChatGPT-4.5 and DeepSeek R1 [64].

Li et al. evaluated the decision-making capabilities of three advanced LLMs (GPT-4o, Claude 3.5, and Grok 2) in endodontic contexts that required specialism. The highest overall accuracy at 73.39% was reached by Claude3.5, followed by Grok 2 at 66.27% and GPT-4o at 46.32%. In the complex case analyses, Grok 2 showed the best performance (69.57%) [65]. In contrast to those studies, the results of the current study showed that ChatGPT-5 produced responses with a higher level of accuracy and completeness than Gemini 2.5 Flash and Claude Sonnet-4, and although Grok 2 showed a similar level of accuracy, it displayed superior performance in terms of completeness. In the HealthBench performance evaluation, which is based on real scenarios and criteria defined by clinicians and published at the start of the year by the manufacturer, ChatGPT-5 significantly outperformed all previous models, obtaining higher points, and showing a significant advance in general capabilities in the field of healthcare [18]. The variability in the results of these different studies suggests that they could be affected by factors such as the version of the model used, the content of the questions asked, sources used as reference, and evaluation criteria.

A previous study systematically evaluated the responses to professional knowledge questions and complex case analysis capabilities of ChatGPT-o3-mini, DeepSeek-R1, Grok-3, Gemini-2.0-Flash-Thinking, and Qwen 2.5-Max models in the field of implant dentistry. They found that the highest overall performance was shown by Gemini 2.0 Flash-Thinking. Both Grok 3 and Qwen 2.5-Max showed similar performances in the responses to professional questions and case analyses, with lower scores obtained than the other three models [5]. The manufacturer has stated that the Grok 4 family clearly outperformed Gemini 2.5 Pro and Claude Opus-4 in most measures of the Graduate-Level Google-Proof Question and Answer (GPQA) science test [28]. Although no significant difference was observed in the current study between Grok 4 and Gemini 2.5 Flash and Claude Sonnet-4, the mean accuracy and completeness scores of Grok 4 were found to be higher. In terms of accuracy, Grok 4 showed a performance similar to that of ChatGPT-5 and obtained worse results in terms of completeness.

In a study by Gurbuz et al., the accuracy and completeness of ChatGPT-4o were evaluated in the treatment of cases with cervical lesions and no decay. A 6-point Likert scale was used to evaluate accuracy, and a 3-point Likert scale was used to evaluate completeness. According to the study results, a statistically significant correlation was found between the degrees of accuracy and completeness in the diagnosis and clinical management areas for ChatGPT-4o [59]. Similarly, in the current study, a significant positive correlation was determined between the accuracy and completeness scores, suggesting that the model has the potential to be a more reliable clinical decision support tool.

Readability refers to the ease of understanding a written text by the reader. Factors such as the intelligence of the reader, education level, environment, areas of interest, purpose and ideas of the text, and the vocabulary, style, and form used can all affect readability. Higher FRESs indicate increased readability of the text, making it easier to understand. In contrast, higher FKGL points, corresponding to education levels in the USA, indicate that a higher level of education is required to understand the text, and readability becomes more difficult [38]. In this context, a strong negative correlation was found between the FKGL and FRES points in the current study. This result shows that FKGL and FRES are complementary criteria, and their evaluation together provides a more holistic view in the evaluation of the readability of a text.

Claude Sonnet-4 has been seen to produce the most complex responses with the lowest median FRES points (8, 3) and the highest median FKGL points (14, 96). These findings showed that the responses of the model contained very complex sentence structures, long phrases, and advanced-level vocabulary, and therefore required an advanced level of academic literacy. Similar to the current study, Sezer et al. examined chatbot performances in response to questions related to traumatic dental injuries in primary teeth, and reported that Claude 3.7 gave the most complex responses, characterized by the lowest FRES and highest FKGL points, requiring advanced level academic literacy [62].

The median FKGL points of 11.7 of ChatGPT-5 correspond technically to the final year of high school. However, the extremely low level of the FRES median points of 22.7 show that the text contained long and complex sentences and required challenging reading at an academic level. The FKGL median points of Gemini 2.5 Flash were higher at 13.1, requiring more advanced literacy in terms of the education level, but the median 31.1 FRES points showed that the text produced was more fluent and relatively understandable compared to ChatGPT-5. In this case, Gemini 2.5 Flash provided more readable responses than other chatbots. Another similar study evaluated the performance of the responses given by AI chatbots to questions frequently asked by patients related to dental prostheses, and Google Gemini was found to have a significantly better performance than the other two chatbots (ChatGPT-3.5 and Microsoft Copilot) [66].

When the FKGL median points of the LLMs were examined in the current study, the highest score of 14.96 points was reached by Claude Sonnet-4, followed by 14.22 points of Grok 4, 13.19 points of Gemini 2.5 Flash, and at 11.81 points, ChatGPT-5 obtained the lowest median points. According to these scores, the texts produced by all the models were in the “difficult” readability category. Similarly, Özcivelek et al. evaluated the accuracy, quality, readability, comprehensibility, and practical applicability of the responses of DeepSeek-R1, ChatGPT-o1, ChatGPT-4, and Dental GPT chatbots; the FKGL points of all the chatbots varied between 9.42 and 10.70, indicating an extremely challenging reading level [38].

The readability of responses to open-ended questions differed markedly across models on all indices (GFI, CLI, and SMOG). Considering that lower scores indicate higher readability, ChatGPT-5 produced more readable texts than Gemini 2.5 Flash, Grok 4, and Claude Sonnet-4 on the GFI; moreover, Grok 4 exhibited higher GFI scores than both Gemini 2.5 Flash and Claude Sonnet-4. For the CLI, ChatGPT-5 yielded lower mean scores than Grok 4 and Claude Sonnet-4, while Gemini 2.5 Flash achieved the lowest CLI values among these three comparators. With respect to SMOG, ChatGPT-5 generated more readable outputs than Gemini 2.5 Flash and Grok 4, whereas Grok 4 showed higher SMOG scores than Claude Sonnet-4. No notable differences were observed in the remaining pairwise comparisons. In a similar vein to our study, Gohari et al. examined the readability levels of LLMs, and ChatGPT received the lowest scores in the GFI and SMOG rankings, followed by Gemini and Claude. In the CLI ranking, however, Gemini obtained the lowest score, followed by ChatGPT and Claude [67]. Furthermore, in the study by Korkmaz et al., which evaluated the readability and accuracy of LLMs, Grok received higher SMOG and CLI scores than Gemini and ChatGPT, thereby producing the most difficult-to-read texts [68]. Overall, these findings indicate that ChatGPT-5 demonstrates a relatively superior profile in terms of readability metrics, whereas Grok 4 tends to generate texts with more challenging readability levels.

Özdemir et al. evaluated the accuracy, reliability, consistency, and readability of the responses of different LLMs related to restorative dental treatment. It was reported that in the AI applications used for dentistry students or in questions specific to dentistry, responses were appropriate to the education level, and no significant readability problems were experienced. However, it was emphasized that it was generally difficult for patients to obtain clear and comprehensible responses to questions about their own health from AI-supported systems. These findings demonstrate that most LLMs produce texts that are difficult to read, and a university-level education may be necessary to be able to fully understand the responses given [69]. Therefore, when the findings of studies are evaluated together, even though questions were asked under the headings of different subjects and different versions of LLMs were used, it can be concluded that the texts produced by LLMs are generally at the level of “difficult” readability. In addition, the complexity of the language used by LLMs can constitute a barrier to comprehensibility and clinical applicability in stressful clinical conditions, such as iatrogenic events in endodontics.

It is remarkable that artificial intelligence can generate explanatory responses within a very short time. This speed and responsiveness may provide dentists, dental interns, and specialists with immediate feedback in cases of iatrogenic events occurring during endodontic procedures, thereby contributing to effective and rational interventions. However, as AI-based large language models (LLMs) are continuously evolving, it is challenging to perform reliable real-time comparisons. Nevertheless, when information is sought regarding an iatrogenic event during endodontic treatment, only instantaneous responses are generally considered clinically meaningful. Therefore, this study presents a real-time analysis in which various AI-based LLMs were asked questions only once and their responses were evaluated accordingly.

AI applications have the potential to develop treatment recommendations for clinical processes in the field of endodontics. However, there is a need for further comprehensive and qualitative scientific studies to be able to consider the benefits that could be provided in practice and the potential risks of these technologies.

## 5. Limitations

This study has several limitations. First, the inclusion of only a limited number of models narrows the scope of the findings. Moreover, the results are specific to the model versions available during the data collection period; future updates may yield different outcomes. The study’s focus on iatrogenic events in endodontics further restricts the generalizability of the findings to other subfields of dentistry. In addition, the evaluations were conducted by only two assessors, no human-generated comparison group was included, and a single reference textbook was used as the sole source. There is still an insufficient number of studies on the most recent versions of the chatbots we have used. Further detailed research should be conducted on the latest versions in relation to other fields of dentistry, and comparative evaluations should be carried out.

## 6. Conclusions

Within the limitations of our study, it was observed that the ChatGPT-5 model generally outperformed other large language models (LLMs) in responding to questions related to iatrogenic events in endodontics. However, while not yet at a level suitable for direct use in clinical practice, there remain areas in need of improvement, particularly with regard to readability and clinical integration. However, the complexity of the language used by LLMs continues to create limitations in terms of comprehensibility and clinical applicability in stressful clinical conditions, such as iatrogenic events in endodontics. This is an important point that must be considered in the direct integration of AI-based solutions in clinical settings. Therefore, further studies are needed to develop AI programs that are more user-friendly and applicable to clinical decision processes and are designed specifically to meet the needs of endodontic clinicians.

## Figures and Tables

**Table 1:** The questions asked to the chatbots.

Questions
What is an iatrogenic event in endodontic treatment?
2. What are the causes of sodium hypochlorite (NaOCl) complications during root canal treatment?
3. What clinical conditions occur in patients when sodium hypochlorite (NaOCl) leaks during root canal treatment?
4. What precautions should a clinician take if they notice an extrusion event of sodium hypochlorite (NaOCl) during root canal treatment? What should the dentist do to treat this condition?
5. What precautions should be taken to prevent sodium hypochlorite (NaOCl) extrusion during root canal treatment?
6. What are the causes of root canal file fracture during root canal treatment?
7. What is cyclic fatigue or torsional fatigue that causes endodontic files to fracture?
8. What should the dentist do to remove the file in the case of a root canal file fracture during root canal treatment?
9. What is the Masserann kit (Micro-Mega, Besançon, France) used for the removal of broken root canal files in endodontics, and how is it used?
10. What are the advantages and methods of using ultrasonics to remove broken root canal files in endodontics?
11. What procedure is followed to remove a broken root canal file if it is visible during root canal treatment?
12. What procedure is followed to remove a broken root canal file if it is in an invisible area during root canal treatment?
13. In which cases should surgical approaches be considered for the treatment of broken root canal files in endodontic treatment?
14. How does the fracture of a root canal file affect the prognosis of endodontic treatment?
15. What is ledge formation in endodontic treatment, and what does it lead to?
16. What are the causes of ledge formation during root canal treatment?
17. What are the methods for avoiding ledge formation in endodontic treatment?
18. In what situations does endodontic-related paresthesia occur?
19. What are the reasons for the extrusion of obturation materials used for root canal filling beyond the radicular foramen?
20. What treatment approaches should be applied to obturation materials used in root canal filling that extend beyond the root apex?
21. What complications may arise from the extrusion of root canal filling material into the maxillary sinus, and what is the role of surgical intervention in managing this condition?
22. What factors cause inferior alveolar nerve damage during endodontic treatment?
23. How should early medical management be conducted to reduce acute nerve inflammation in endodontics? Which medications should be used?
24. What are the causes of cervical subcutaneous emphysema in endodontic treatment?
25. What treatment approach should be adopted when cervical subcutaneous emphysema occurs during endodontic treatment?
**Total** 25 questions

**Table 2:** Interpretation of the Flesch Reading Ease Score (FRES) and Flesch–Kincaid Grade Level (FKGL).

FRES	Reading Level	FKGL	Estimated Reading Graduate Level
90–100	Very Easy	5	5th Grade
80–89	Easy	6	6th Grade
70–79	Fairly Easy	7	7th Grade
60–69	Standard	8–9	8th–9th Grade
50–59	Fairly Difficult	10–12	High School
30–49	Difficult	13–16	College Level
0–29	Very Difficult	>16	College Graduate

**Table 3:** Comparisons of the accuracy, completeness, and readability scores of the ChatGPT-5, Gemini 2.5 Flash, Grok 4, and Claude Sonnet-4 models.

		Chatbot				*p* Value
		ChatGPT-5	Gemini 2.5 Flash	Grok-4	Claude Sonnet-4	
Accuracy	Mean ± SD	4.56 ± 0.65	3.64 ± 0.95	3.96 ± 1.09	3.44 ± 1.19	0.001 ^a^
	Median (min.–max.)	5 (3–5)	3 (2–5)	4 (2–5)	3 (1–5)	
Completeness	Mean ± SD	2.88 ± 0.33	2.24 ± 0.52	2.28 ± 0.61	1.68 ± 0.75	<0.001 ^a^
	Median (min.–max.)	3 (2–3)	2 (1–3)	2 (1–3)	2 (1–3)	
Readability						
FKGL	Mean ± SD	11.81 ± 2.04	13.19 ± 1.95	14.22 ± 2.76	14.96 ± 2.15	<0.001 ^a^
	Median (min.–max.)	11.7 (8.6–15.9)	13.1 (10.2–16)	13.9 (10.1–20.5)	15.1 (10.8–19.5)	
FRES	Mean ± SD	25.79 ± 14.27	32.09 ± 10.73	18.93 ± 14.26	11.08 ± 12.16	<0.001 ^a^
	Median (min.–max.)	22.7 (4.1–48.9)	31.1 (12.9–48)	19 (0–42.9)	8.3 (0–35.8)	
GFI	Mean ± SD	13.16 ± 2.4	15.37 ± 2.09	17.51 ± 2.68	15.56 ± 2.26	<0.001 ^b^
	Median (min.–max.)	12.5 (9.9–18)	14.5 (12.1–19.1)	17.3 (13–23.2)	15 (11–21.4)	
CLI	Mean ± SD	15.14 ± 2.15	13.82 ± 1.19	17.15 ± 1.68	18.24 ± 1.67	<0.001 ^b^
	Median (min.–max.)	15.7 (10.9–19.2)	13.7 (11.9–16.2)	17.3 (14.1–19.8)	18.3 (14.8–21.2)	
SMOG	Mean ± SD	13.15 ± 1.49	15.06 ± 1.44	16.75 ± 1.99	14.35 ± 1.78	<0.001 ^a^
	Median (min.–max.)	13.1 (11.1–17)	14.3 (13–17.3)	16.1 (13.7–20.4)	14.2 (11.4–18.9)	

The data are expressed as mean ± standard deviation and median (minimum: maximum). ^a^: Kruskal–Wallis test, ^b^: ANOVA test.

**Table 4:** Spearman correlation analysis between the accuracy, completeness, and readability scores for ChatGPT-5, Gemini 2.5 Flash, Grok 4, and Claude Sonnet-4.

	Accuracy	Completeness
Completeness		
r_s_	0.77	-
*p* value	<0.001	-
FKGL		
r_s_	−0.13	−0.19
*p* value	0.186	0.047
FRES		
r_s_	0.02	0.08
*p* value	0.835	0.453
GFI		
r_s_	−0.10	−0.19
*p* value	0.313	0.057
CLI		
r_s_	−0.01	−0.15
*p* value	0.890	0.129
SMOG		
r_s_	−0.15	−0.20
*p* value	0.136	0.045

r_s_: Spearman’s correlation coefficient.

**Table 5:** Pearson correlation analysis between readability scores for ChatGPT-5, Gemini 2.5 Flash, Grok 4, and Claude Sonnet-4.

	FKGL	FRES	GFI	CLI	SMOG
FRES					
R	−0.88	-	-	-	-
*p* value	<0.001	-	-	-	-
GFI					
r	0.81	−0.59	-	-	-
*p* value	<0.001	<0.001	-	-	-
CLI					
r	0.76	−0.89	0.58	-	-
*p* value	<0.001	<0.001	<0.001	-	-
SMOG					
r	0.72	−0.45	0.94	0.46	-
*p* value	<0.001	<0.001	<0.001	<0.001	-

## Data Availability

The original contributions presented in this study are included in the article. Further inquiries can be directed to the corresponding author.

## Abbreviations

The following abbreviations are used in this manuscript:

AI	Artificial Intelligence
LLMs	Large Language Models
FRES	Flesch Reading Ease Score
FKGL	Flesch–Kincaid Grade Level
GFI	Gunning Fog Index
SMOG	Simplified Measure of Gobbledygook
CLI	Coleman–Liau Index
